# The uncharted territory of female adult ADHD: a comprehensive review

**DOI:** 10.1192/j.eurpsy.2024.624

**Published:** 2024-08-27

**Authors:** B. A. Oroian, G. Costandache, E. Popescu, P. Nechita, A. Szalontay

**Affiliations:** Institute of Psychiatry “Socola” Iasi, Iasi, Romania

## Abstract

**Introduction:**

Attention-Deficit/Hyperactivity Disorder (ADHD), once considered a predominantly childhood condition, has increasingly gained recognition as a prevalent and clinically significant concern among adult women. They often display a distinctive symptom profile characterized by high levels of inattention, emotional dysregulation, and difficulties in executive functioning. Diagnosis of female adult ADHD is frequently complicated by gender bias in traditional diagnostic criteria, which may fail to account for the unique ways in which women manifest the disorder.

**Objectives:**

This comprehensive literature review aims to characterize the unique symptomatology of female adult ADHD, including variations in inattention, hyperactivity, and impulsivity, as well as the presence of emotional dysregulation. It also seeks to explore the diagnostic challenges stemming from gender bias in diagnostic criteria and the role of comorbidity in diagnostic complexity. Additionally, the review assesses the broad spectrum of functional impairments experienced by adult women with ADHD, spanning academic, occupational, interpersonal, and emotional domains.

**Methods:**

This literature review comprises a systematic examination of published research articles, clinical studies, and relevant academic literature addressing female adult ADHD. A comprehensive search strategy involving electronic databases, including PubMed, PsycINFO, and Google Scholar, was employed to identify peer-reviewed articles published between 2000 and 2023. The selected studies underwent critical appraisal for quality and relevance to the review’s objectives.

**Results:**

The synthesis of existing literature reveals that female adult ADHD presents a distinctive clinical picture characterized by a higher prevalence of inattention, emotional dysregulation, and comorbid conditions such as mood and anxiety disorders. Diagnostic challenges arise from gender bias in diagnostic criteria and the absence of overt hyperactivity, often leading to delayed diagnosis or misdiagnosis. Functional impairments extend to academic, occupational, interpersonal, and emotional domains, affecting the overall quality of life for affected individuals. Gender-specific factors, including societal expectations and biases in healthcare evaluation, contribute to diagnostic disparities and hinder timely access to appropriate interventions.

**Conclusions:**

The literature review underscores the critical need for enhanced recognition, understanding, and tailored support for female adults with ADHD. The distinct symptomatology, diagnostic complexities, functional impairments, and gender-specific factors contribute to a multifaceted clinical landscape. Advancing gender-sensitive diagnostic criteria, increasing awareness among healthcare professionals, and developing interventions that address the unique needs of this population are essential steps toward improving the quality of life and outcomes for female adults with ADHD.

**Disclosure of Interest:**

None Declared

